# Supplementary figures and images for: The impact of HTLV-1 expression on the 3D structure and expression of host chromatin
Source: PLoS Pathog. 2024 Mar 1;20(3):e1011716. doi: 10.1371/journal.ppat.1011716 (PMC10936777; doi:10.1371/journal.ppat.1011716)

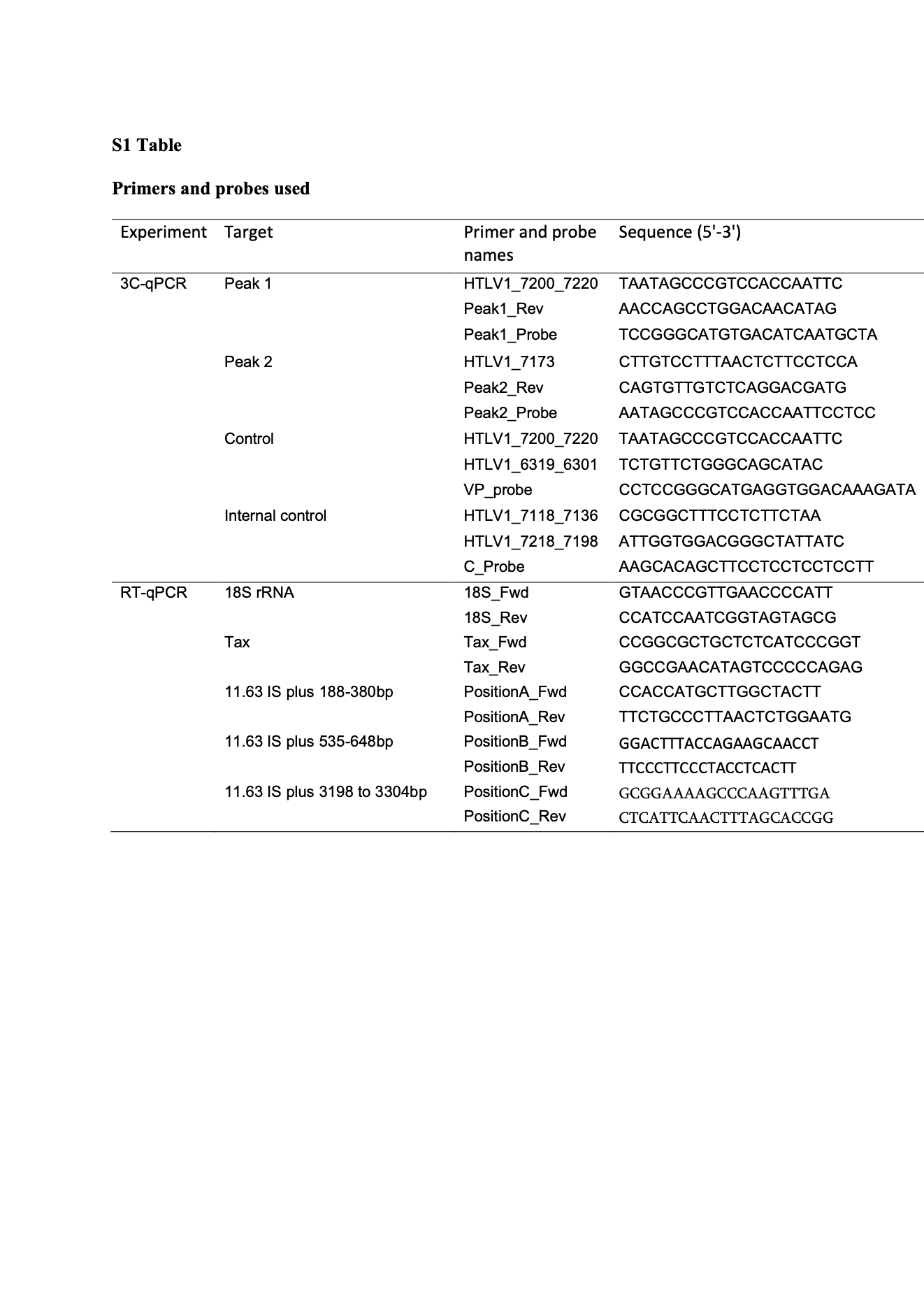

Supplement: S1 Table — (TIFF) [file ppat.1011716.s001.tiff]

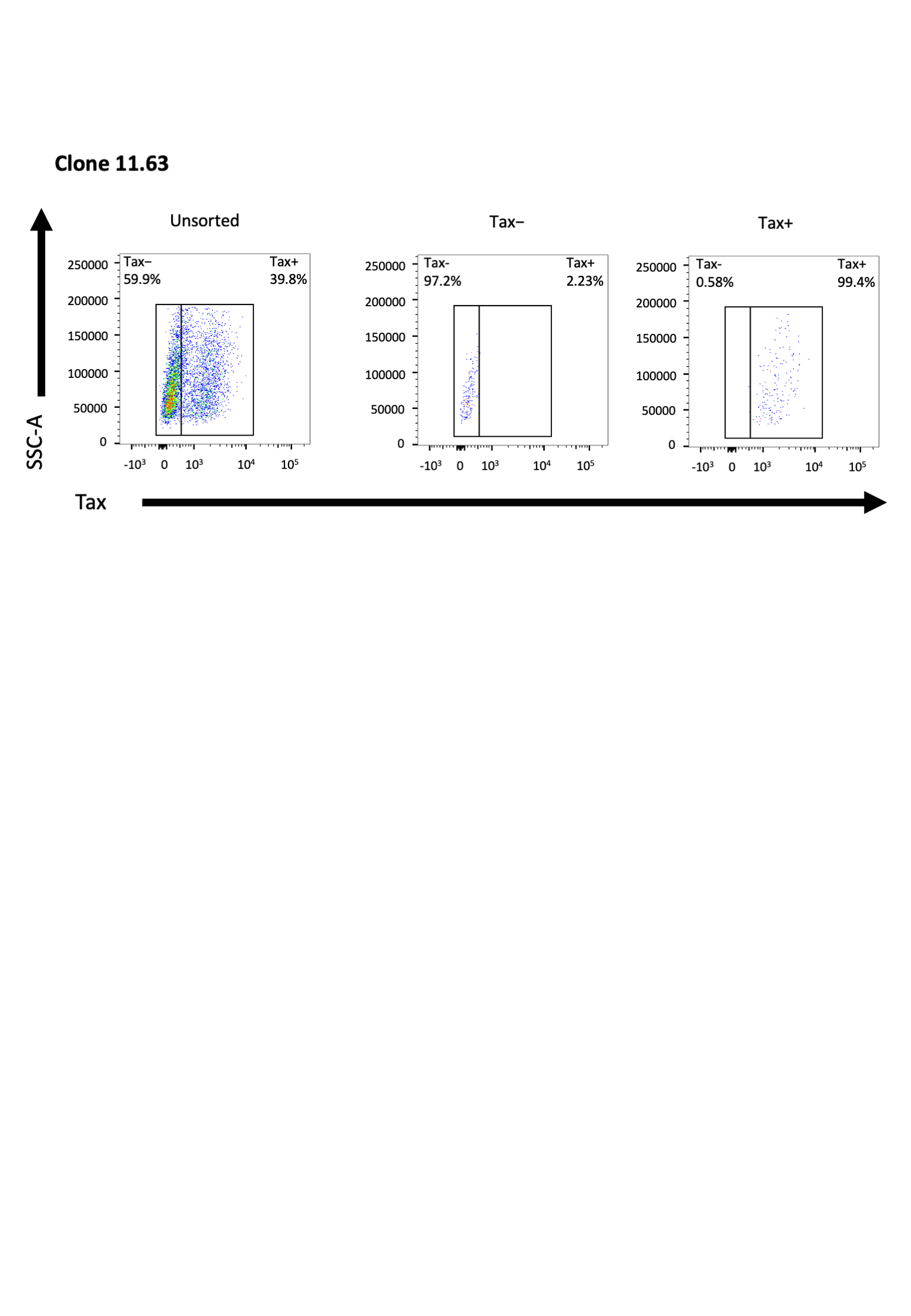

Supplement: S1 Fig — HTLV-1-infected T cell clone 11.63 was stained for live cells, crosslinked in 1% formaldehyde, stained intracellularly for Tax protein, and flow-sorted to isolate Tax−and Tax+ subsets (see Methods). (TIFF) [file ppat.1011716.s002.tiff]

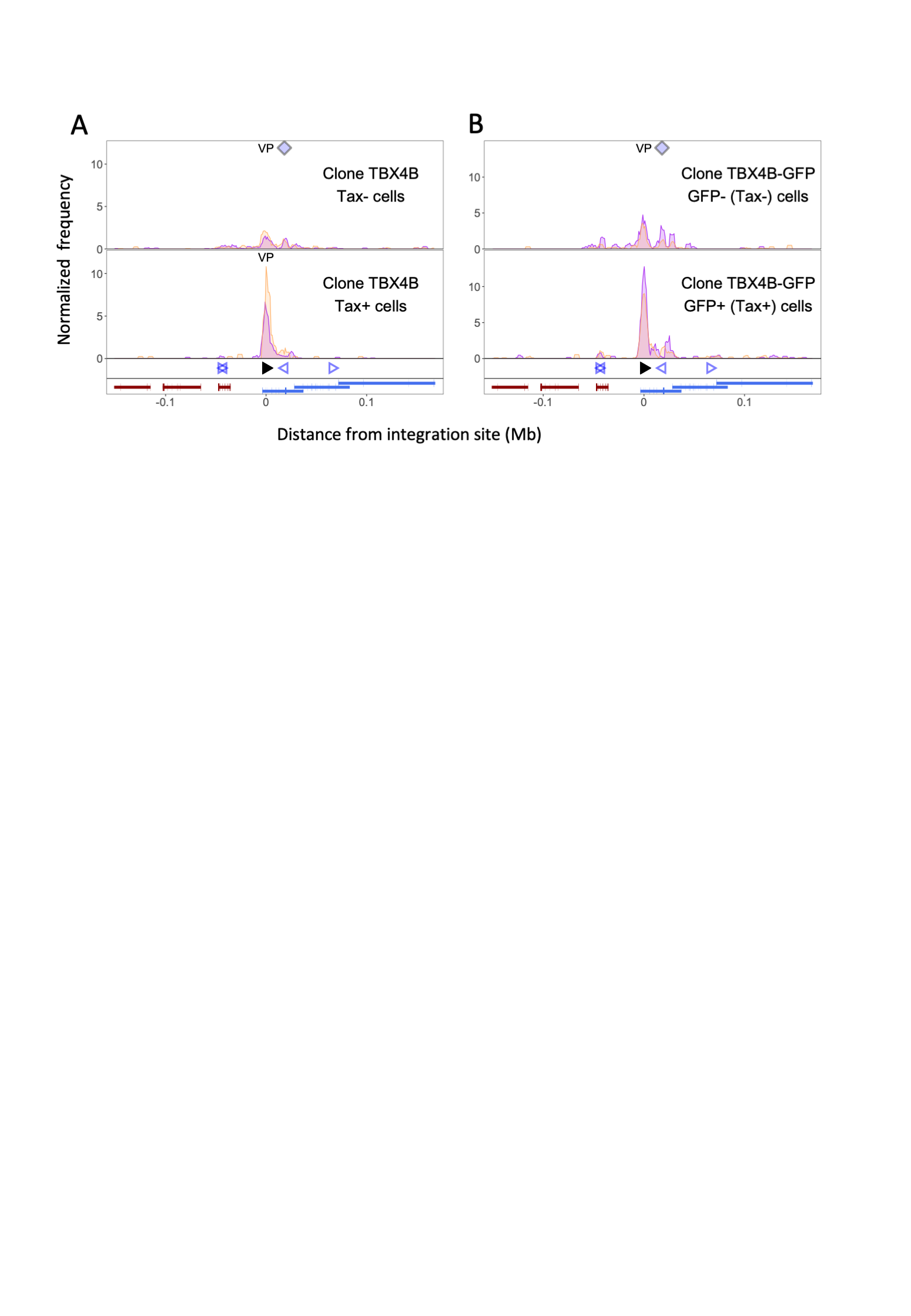

Supplement: S2 Fig — (A) q4C profiles of Tax−(upper panel) and Tax+ (lower panel) cells sorted from clone TBX4B after intracellular staining of Tax. (B) q4C profile of non-expressing (GFP-) (upper panel) and provirus-expressing (GFP+) (lower panel) cells isolated from d2EGFP-TBX4B clones, selected by GFP signal (without Tax staining). (TIFF) [file ppat.1011716.s003.tiff]

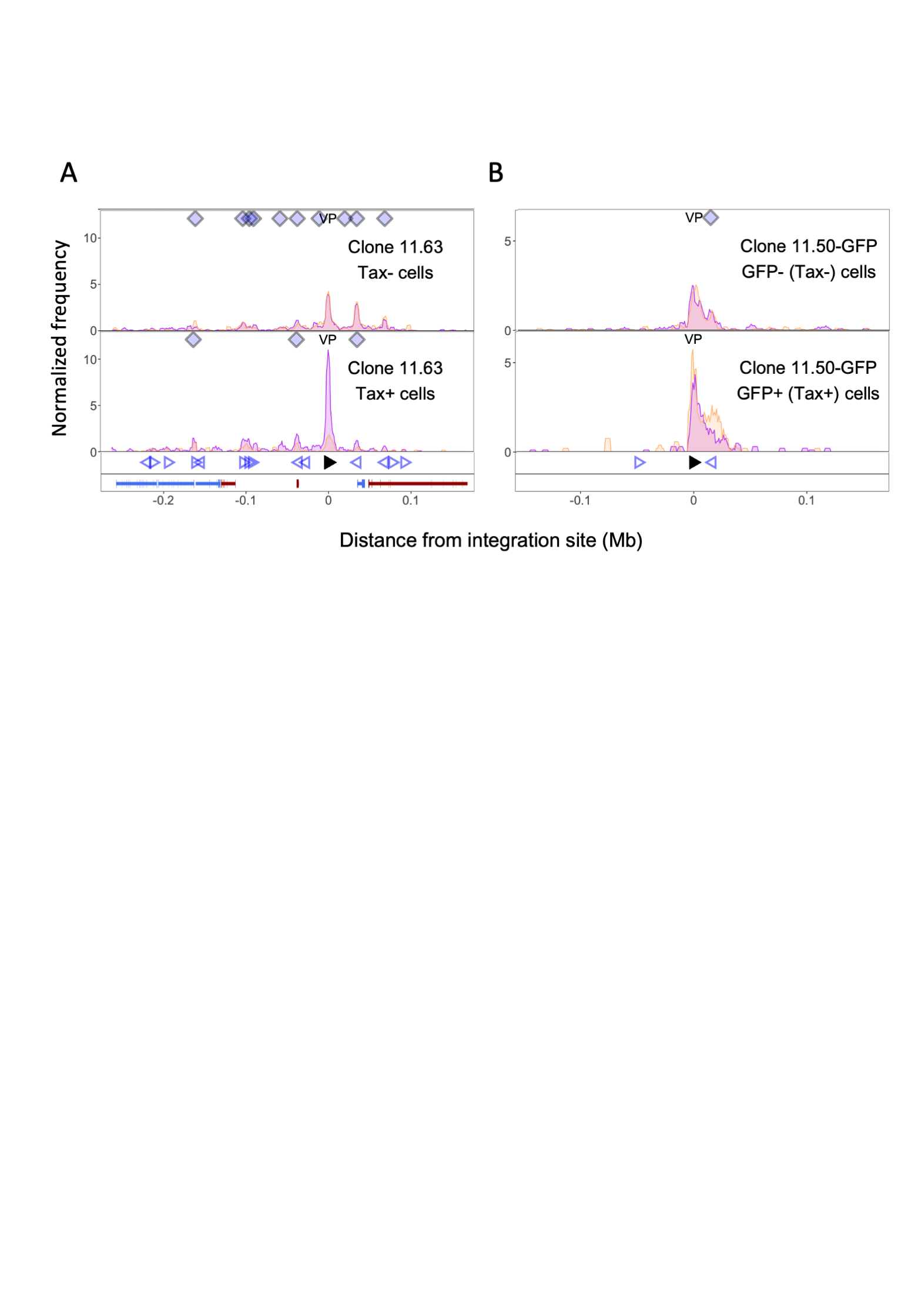

Supplement: S3 Fig — (A) q4C profiles of Tax−(upper panel) and Tax+ (lower panel) cells from clone 11.63. (B) q4C profiles of non-expressing (GFP–) (upper panel) and provirus-expressing (GFP+) cells (lower panel) from clone d2EGFP-11.50. Vertical axes show the normalized frequency of chromatin contacts between the provirus and the host genome. (TIFF) [file ppat.1011716.s004.tiff]

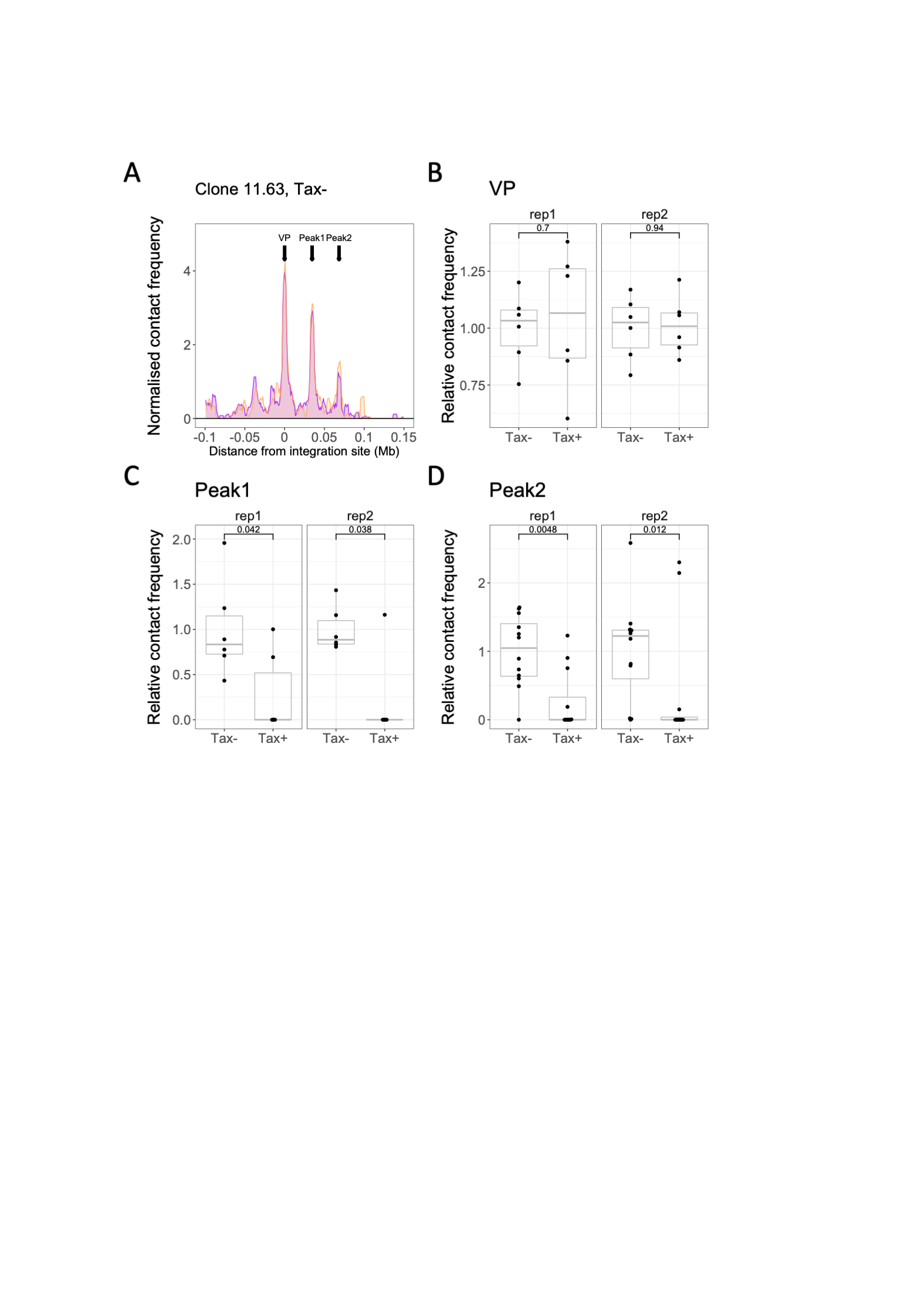

Supplement: S4 Fig — (A) q4C profile of Tax−cells of clone 11.63. The technical peak seen in the q4C viewpoint (VP) and two of the main peaks (Peak 1) and (Peak 2) identified in the output of the Tax−fraction of clone 11.63. (B) As control, the frequency of chromatin interactions was quantified by 3C-qPCR on sorted Tax−and Tax+ cells, using a primer pair and Taqman probe to detect the contacts between two regions: VP and another region in the provirus (S1 Table). There was no significant difference between Tax+ and Tax−cells (combined p value = 0.932, Fisher’s method of combining p values). (C and D) Primer pairs and probe were used to detect long-range chromatin contacts between the provirus and host genome region at Peak 1 (C) or Peak 2 (D). Results of 3C-qPCR of two biological replicates (rep) are shown. Peak1 contact frequency was significantly higher in Tax−cells than in Tax+ cells (combined p value 0.012, Fisher’s method). Peak2 contact frequency was significantly higher in Tax- cells than in Tax+ cells (combined p value 0.000607, Fisher’s method). (TIFF) [file ppat.1011716.s005.tiff]

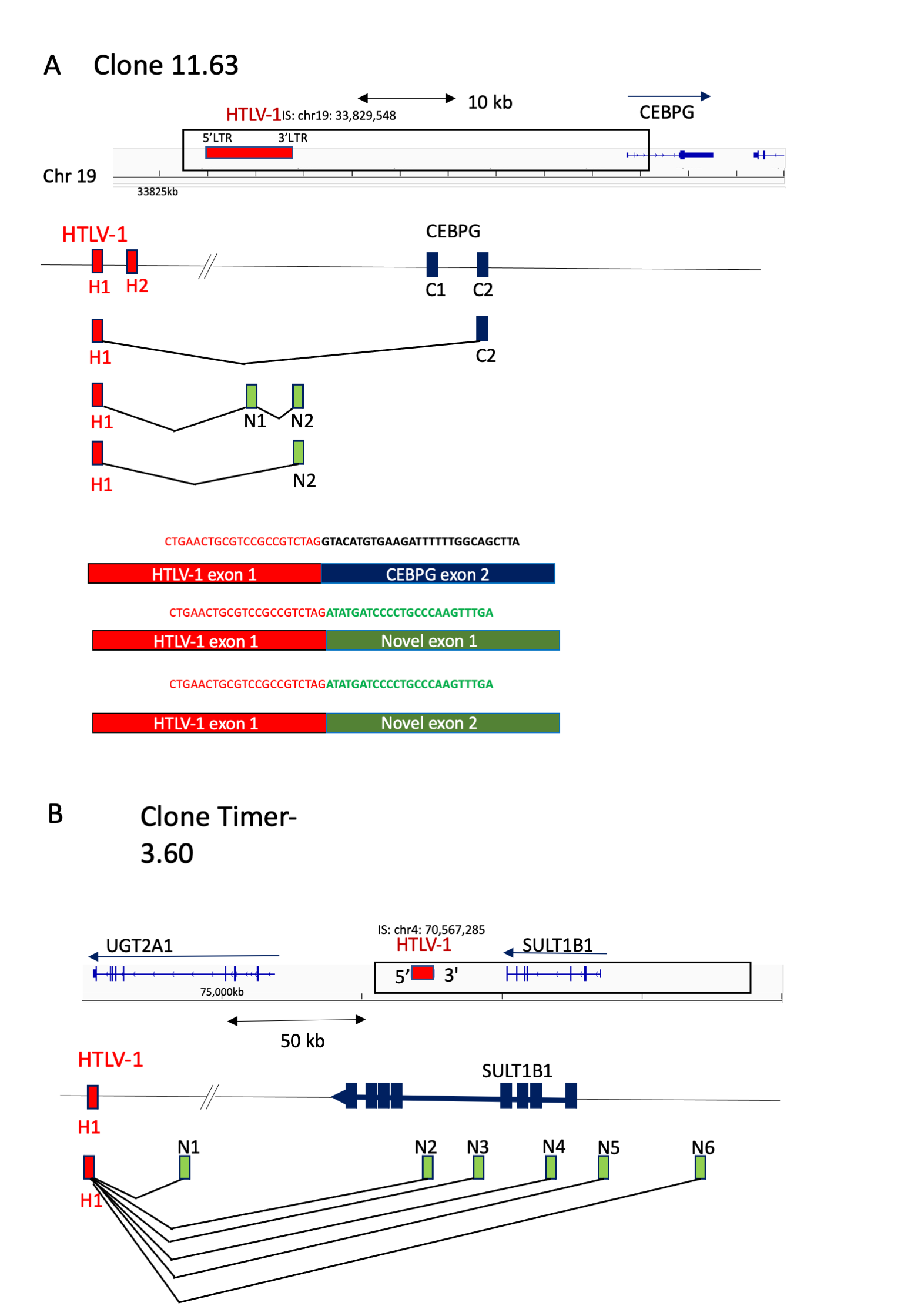

Supplement: S5 Fig — (A) Identification of splice sites of fusion transcripts in the plus-strand-expressing cells of clone d2EGFP-11.63 and (B) in timer protein reporter clone Timer-3.60. Plus-strand fusion transcripts between HTLV-1 exon1 (H1) and same sense, 3′ side host gene exon (blue) or novel host exons (green) are shown with fused sequences. (TIFF) [file ppat.1011716.s006.tiff]

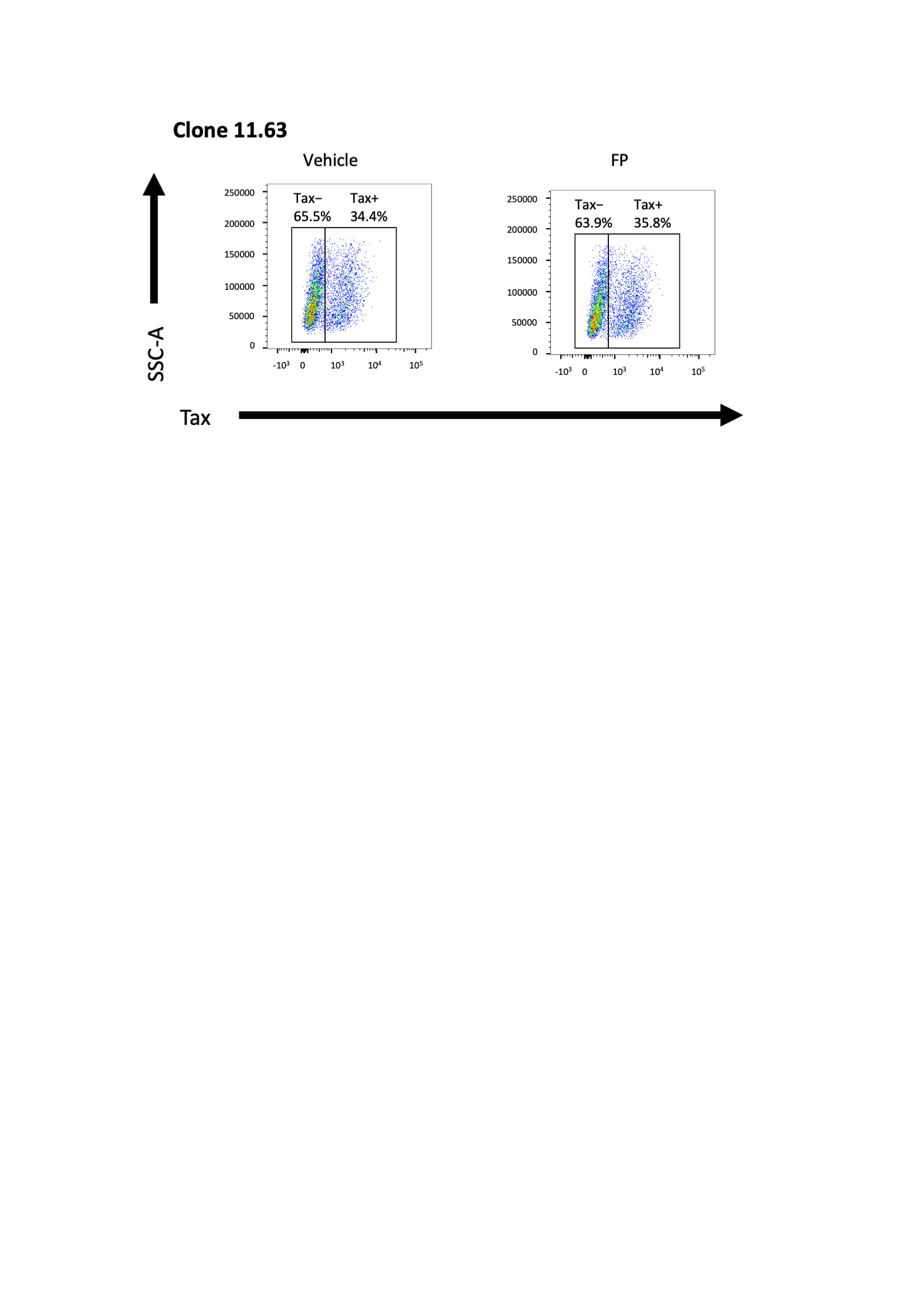

Supplement: S6 Fig — Clone 11.63 cells were treated with 1nM flavopiridol for 1.5 hrs and then stained for Live/Dead and then Tax protein. (TIFF) [file ppat.1011716.s007.tiff]

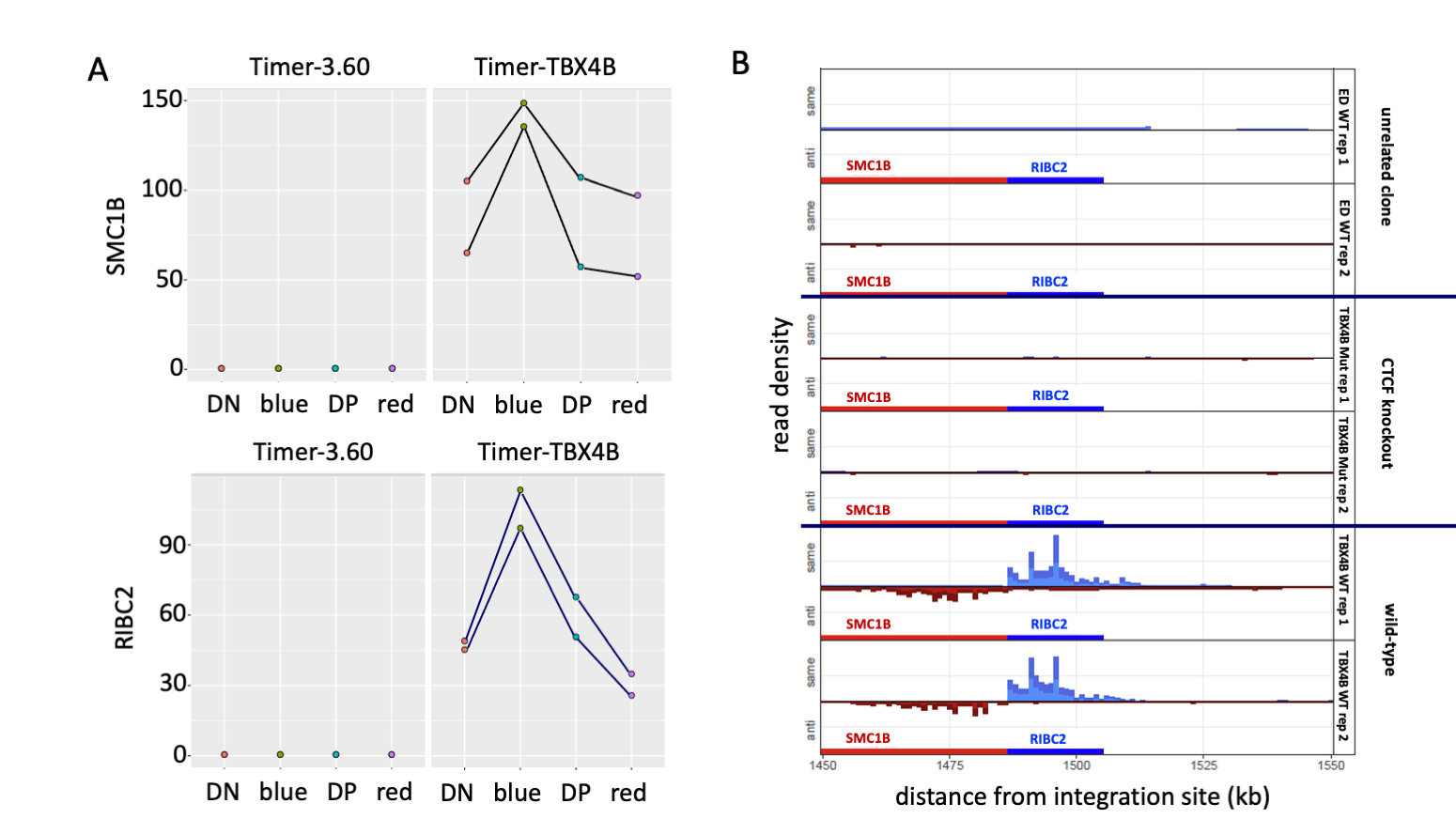

Supplement: S7 Fig — (A) Normalized mRNA read counts of two genes that lie >1.4 Mb from the provirus in clone Timer-TBX4B, in the four successive phases of the HTLV-1 plus-strand transcriptional burst: DN–double negative (HTLV-1 silent); blue–early burst; DP double-positive (mid-burst); red–late burst. Results of two independent experiments are shown. Expression of both SMC1B and RIBC2 closely followed the trajectory of the HTLV-1 burst in clone Timer-TBX4B, but not in the unrelated HTLV-1-infected clone Timer-3.60. Data from [11]. (B) Knockout of the CTCF binding site in the provirus in clone Timer-TBX4B (middle panel) abolished the transcription of both SMC1B and RIBC2 observed in the wild-type clone (lower panel). Results of two independent experiments are shown. Neither gene was expressed in an unrelated HTLV-1-infected clone ED. The results suggest that maintenance of a CTCF-dependent chromatin loop between the host genome and the provirus is required for the burst of transcription of these distant genes associated with the HTLV-1 plus-strand burst. (TIFF) [file ppat.1011716.s008.tiff]

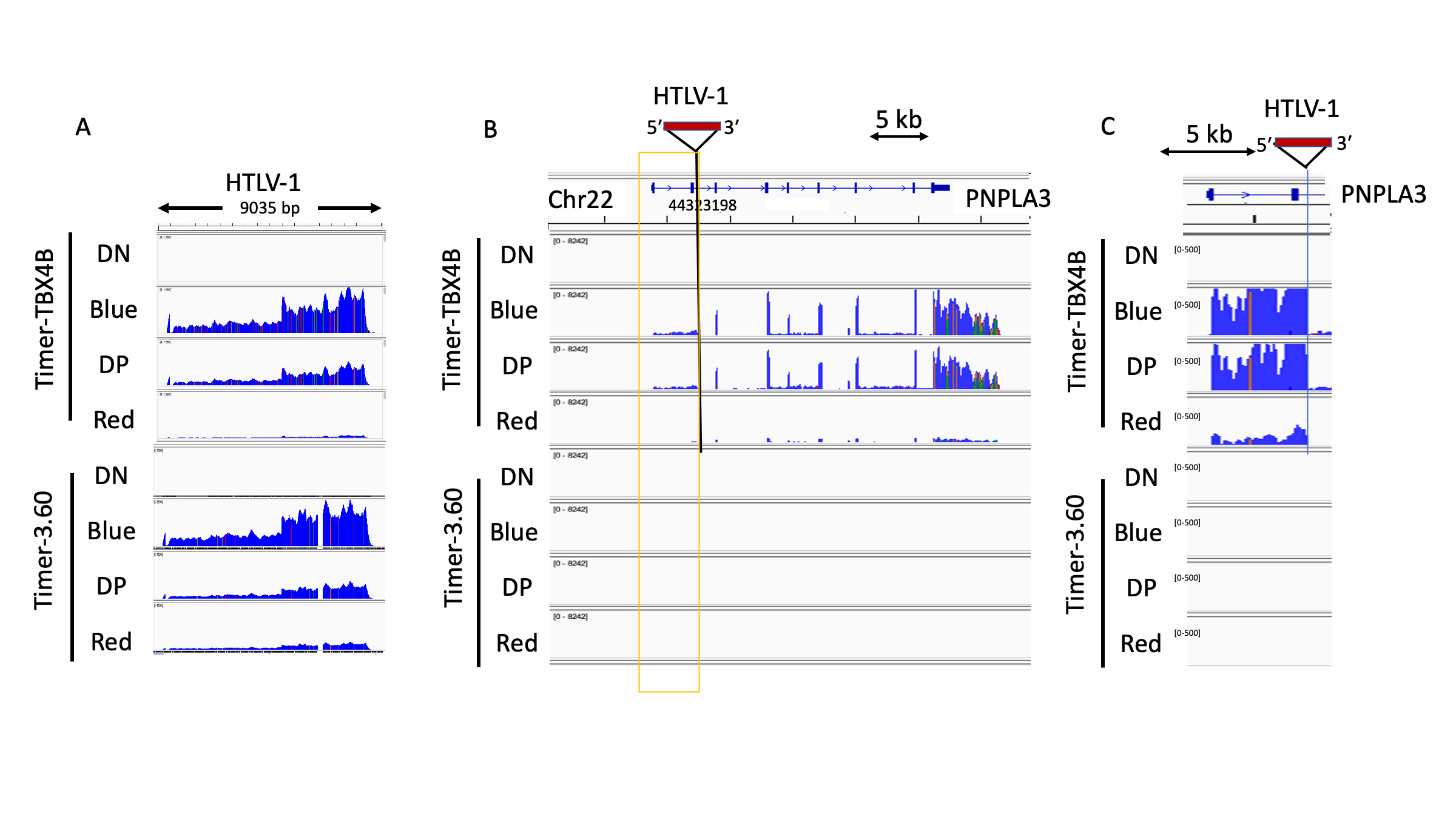

Supplement: S8 Fig — Cells were sorted into four populations based on the fluorescence of the Timer protein, DN–double negative (HTLV-1 silent); blue–early burst; DP double-positive (mid-burst); red–late burst. [11].(A) Coverage tracks in IGV of plus strand HTLV-1 provirus transcription and (B) host gene PNPLA3 transcription. (C) Transcription in PNPLA3 exons 1 and 2 (note range on vertical axis 0 to 500). (TIFF) [file ppat.1011716.s009.tiff]
