# Supplementary material for: The impact of HTLV-1 expression on the 3D structure and expression of host chromatin
Source: PLoS Pathog. 2024 Mar 1;20(3):e1011716. doi: 10.1371/journal.ppat.1011716 (PMC10936777; doi:10.1371/journal.ppat.1011716)
Supplement: Attachment — Submitted filename: 240108 - Yaguchi et al - response to reviewers.docx [file ppat.1011716.s010.docx]

**Yaguchi et al. -** **The impact of HTLV-1 expression on the 3D structure and expression of host chromatin**

**Response to reviewers’ comments:**

Reviewer #1: Major Concerns
**1) The graphs in Fig 2A are missing. The numbers of peaks listed in Fig 2B are very small and inadequate for any firm conclusions.**

Please find Fig 2A and 2B amended and uploaded properly.

**2) Almost all of the results are from subjects with HAM/TSP. Are these results unique to this subset?**

We previously showed that HTLV-1 forms chromatin loops with the host genome in clones from ACs (asymptomatic carriers) (Melamed, Yaguchi et al. 2018). Further, there is no systematic difference in the proviral integration site between clones isolated from ACs and patients with HAM/TSP (Niederer, Laydon et al. 2014). The only reproducibly identified difference in the infected T cell clones between HAM patients and ACs lies in the total number of clones (which are higher in HAM), not in the individual characteristics of the clones. We conclude that it is highly unlikely that these results are unique to HAM/TSP.

**3) Possible impact of fixation, flow sorting, or transduction of reporters on the transcription results obtained.**

Fixation in 1% paraformaldehyde is an essential step in the q4C assay, as in 3C and other ‘C’ assays; its effects on chromatin looping therefore cannot be directly tested. Because fixation can result in low quality of RNA, we only analysed RNAs extracted from unfixed cells.
S2 Fig shows a similar 4C profile from transduced and non-transduced cells. Therefore, since chromatin looping depends on the genomic position of the provirus (Melamed, Yaguchi et al. 2018), we infer that transduction per se does not affect q4C analysis. We have added a sentence to this effect to the Results section. There was no systematic difference in transcription between transduced and normal clones in our previous study (Melamed, Yaguchi et al. 2018).

**Possible impact of Tax expression on chromatin looping and transcription.**

We addressed this point in the previous study (Melamed, Yaguchi et al. 2018), in which we included clones that did not express Tax. In addition to the chromatin loops formed between the provirus and the host genome, we showed that the provirus *per se* caused aberrant transcription, mainly antisense on the 5′ side of the provirus, which is consistent with the results obtained in the present study.

**4) Why does transcription affect chromatin contacts outside the provirus but not within the provirus?**

The experiment was designed to answer the question whether the contacts between the provirus and the flanking host genome changed with proviral transcription. The individual positions and frequencies of ‘internal’ loops within the provirus could not be precisely quantified, since the contact points are very close to the 4C viewpoint, below the resolution of the assay.

**5) The changes in 4C profile in Fig 5C with flavopiridol are subtle and limited to a single cell line.**

This experiment requires a large number of cells--at least 30-60 million cells before sorting. The growth of these primary cell clones is slow, and sufficient cells for this experiment were obtained only for clone 11.63. However, the results using 11.63 were reproducible in biological replicate experiments. The change in the number of peaks was identified using a three-state hidden Markov model, as previously reported (Melamed, Yaguchi et al. 2018): see Materials and Methods.

**Why did flavopiridol not decrease Tax mRNA levels?**

The duration of treatment with the elongation inhibitor flavopiridol (1.5 h) was too short to significantly reduce the abundance of pre-existing *tax* mRNA but was long enough to reduce readthrough transcription. Our results closely parallel the previous observations made by Heinz et al. on transcription in flavopiridol-treated, influenza virus-infected cells (Heinz, Texari et al. 2018).

**Reviewer #2: 1) Figure 3 is missing some information. Are we looking at all the q4C peak regions of all cell clones analyzed? The genomic position (distance from integration site) is missing numbers (Mb) to denote the positions analyzed.**

Yes: we looked at all peaks in all clones and we have added a sentence explaining this to the legend. Fig 3A is a schematic diagram; the units (Mb) are therefore not shown. We have clarified the legend to explain the method of quantification of q4C peaks in the Tax-expressing and non-Tax expressing cell populations.

2) **Figure 5 – apparent discrepancy between HTLV-1 sense transcription and 3’ host sense transcription, since the elongation inhibitor does not significantly affect Tax mRNA.**

The aim of this study was to see if readthrough transcription, rather than Tax expression itself, decreased chromatin loop formation. As noted above (Reviewer 1, point 5), 1.5 h treatment of this elongation inhibitor was too short to significantly reduce the abundance of pre-existing *tax* mRNA but was long enough to reduce readthrough transcription. Our results closely parallel the previous observations made by Heinz et al. on transcription in flavopiridol-treated, influenza virus-infected cells (Heinz, Texari et al. 2018).
 **3) Effect of sense transcription/chromatin changes on transcription at distal host genomic sites.**

We show here evidence (S7 Fig) that the provirus can affect transcription of host genes within 2 Mb, and that readthrough transcription declines rapidly away from the provirus. We have also previously reported (Melamed, Yaguchi et al. 2018) that the frequency of chromatin loops decreases to low levels beyond 5 Mb of the provirus. It is therefore unlikely that provirus-induced looping will significantly alter the expression of still more distant host genes.

**4) Clarification of S7 Fig and S8 Fig in the text.**

Our observation provides proof of concept that alteration of chromatin looping by the provirus may affect the transcription of distant host genes.

We have added a sentence in the Discussion section to the paragraph describing the results in S7 Fig, to explain the putative reason for this observation. We think the observation is of interest, but it is beyond the scope of the present study to pursue it in detail.

**Reviewer #3: 1. The chromatin loops formed by the proviral DNA involve only host DNA sequences that are adjacent to the integration sites and syntenic. Is this because of the constraint of the q4C technique or the nature of chromatin loops in general? The authors should discuss these points in more depth.**

We have added this to the Discussion as follows:

The chromatin loops formed by the provirus reported here and previously (Melamed, Yaguchi et al. 2018) are made with adjacent regions of the host genome. The frequency of these loops declines rapidly within ~5 Mb of the provirus. These observations are consistent with the size distribution of normal chromatin loops in the human genome (Kempfer and Pombo 2020), and with the mechanism of loop extrusion by which these loops are formed (Davidson and Peters 2021). It is known that contacts can also be made with more distant locations on the genome, including other chromosomes; however, such distant contacts are typically much less frequent, and vary between individual cells, although certain trans-chromosomal contacts may be evolutionarily conserved (Lohia, Fox et al. 2022).

**2. It is reported that there are between 15000 and 40000 CTCF binding sites in the human genome. Do most chromatin regions that HTLV-1 proviral DNA interacts with (as mapped in this study) contain one or more CTCF sites?**

Yes: this was shown in our previous study (Melamed, Yaguchi et al. 2018).

**3. It should be pointed out to the readers that the so-called aberrant host transcription and splicing observed is primarily due to mRNA transcriptional readthrough and splicing (Fig. 4, Fig. S5). This is well known for retroviral transcription, and HTLV-1 is no exception.**

Thank you for this suggestion. We have clarified this point by adding the sentence “Aberrant read-through transcription and splicing is known for retroviral transcription.” in the Discussion section, with a reference to *Retroviruses*, (eds. Coffin et al.)

**4. The TBX4B study (Fig. S7) suggests the long-range “enhancer” effect of HTLV-1 requires the CTCF site and active viral transcription (with the recruitment of Tax-CREB-CBP/p300?). Is this observed in other HTLV-1-infected cell clones in this study?**

This is a very interesting point. Since each clone’s proviral integration site is unique, it is likely that the provirus will lie within an accessible genomic distance (~5 Mb) of both a host CTCF binding site and a gene that may be activated only in a minority of clones. Our present observations provide proof of principle, but it will be necessary to study a large number of such clones to determine the frequency with which this phenomenon occurs.

We have added a sentence to the Discussion section as follows: “This result (S7 Fig) suggests the long-range “enhancer” effect of HTLV-1 requires the CTCF binding to the provirus and active viral transcription. Further investigation will be needed to clarify this mechanism.”.

**Part III – Minor Issues**

Reviewer #1: Minor Concerns
**6) In Table 1, if the designations of subjects are patient initials they should be removed. Also, it is not clear to what the provirus orientation is compared – the centrosome?**

The designations of patients are a code, which doesn't include any patient's personal information. No patient’s initials or other means of identification are included in the paper.

Regarding the orientation, all observations in the present study are reported by reference to the orientation of the provirus itself. We have clarified this in the legend to Table 1.

**7) Table 2 should be combined with Table 1. Why did they not look at Tax expression in all the cell clones? Why were so many cells positive in 3 cell lines (36-56% of cells) when the text says that “a small proportion of cells express intense bursts of Tax.”**

The reporter clones (GFP or Timer) shown in Table 1 were established from clones shown in Table 2. The proportion of cells expressing endogenous Tax in reporter clones was the same as in these clones when they were established ((Kiik, Ramanayake et al. 2022, Ramanayake, Moulding et al. 2022)). We have added a note to this effect in the legend to Table 2.

**High proportion of Tax protein-expressing cells**. Thank you for raising this point. Each infected cell expresses uncommon but intense bursts of transcription of *tax* (Billman et al 2017). However, the Tax protein persists for some days in each cell (Ramanayake et al. 2022). In the present experiment, the cells were selected on the basis of Tax protein expression: at any one time, therefore, the percentage of Tax protein-positive cells at any instant in these clones in vitro is relatively high. The Tax+ population therefore contains both cells that are still expressing *tax* mRNA and those in which Tax protein outlasts the mRNA. It is therefore likely that the reduction in chromatin looping observed here is still greater in cells that are actively expressing the mRNA. This point is now explained in the legend to Table 2.

8) **Fig S2 lacks a label on the x-axis. Why are the patterns so different in the top and bottom figures of each of S2 Fig and S3 Fig which presumably are replicate analyses?**

We apologize for the omission: the x-axis has now been labelled on Fig S2.

The top and bottom panels in S2 Fig and S3 Fig each show the frequency of q4C peaks in Tax-negative cells and Tax-positive cells, respectively: this is shown on each respective panel.

**9) Fig 3A is unclear – is this just a schematic without actual data? why are there 2 peaks downstream of the provirus and only 1 upstream? It is surprising that there are so manyt contact sites without CTCF sites. In Fig 3 legend what is meant by “….both in both peaks with a CTCF site…” In Fig 3C, what are each of the panels?**

Thank you for the comment. We have clarified the legend to the schematic diagram in Fig. 3A to explain the method of quantification of q4C peaks in the Tax-expressing and non-Tax expressing cell populations. Typically, there are more frequent q4C peaks downstream of the HTLV-1 provirus than upstream (Melamed, Yaguchi et al. 2018). This is because the proviral CTCF binding site is oriented forwards (5′ to 3′), and it is well established that chromatin loops usually form between two CTCF molecules in convergent orientation. The following sentence has been added to the Fig 3C legend: “Peak height was calculated in each peak region for peaks with a CTCF site and those without CTCF sites.”

The phrase **“**both in both peaks” was corrected to **“**both in peaks”.

**10) How were the genes selected in S7 Fig for analysis? Can these results be generalized?**
The genes were not selected, but were empirically observed on the basis of the abundance of mRNA in either the CTCF-BS knockout or the wild-type cells (S7 Fig B); we subsequently confirmed dysregulation of the genes observed (S7 Fig A) using the Timer protein reporter (Kiik, Ramanayake et al. 2022).

Reviewer #2: 1) **The authors show that read-through of same sense transcription in the host genome could lead to production of aberrant mRNAs. They then comment in the Discussion that read-through of same sense transcription in the host genome was rarely observed in other previous studies using ATL cells (Kataoka et al., Rosewick et al.). Could these observations be interpreted as infected clones that produce aberrant mRNAs from read-through, same-sense transcription being selected against becoming ATL cells?**

This is a very interesting and perceptive question. It is well established that plus-strand expression is reduced and indeed frequently silenced in ATL clones, by deletion, mutation or DNA methylation (Takeda et al. 2004: Int. J. Cancer 109, 559). It is widely assumed that this silencing is the result of immune-mediated selection against the plus-strand-encoded antigens – principally Tax protein. Consequently, readthrough transcription is less commonly observed in such clones. ATL clones appear to be driven by a combination of persistent *HBZ* expression from the minus strand of the provirus and oncogenic mutations in the host genome.

**2) Additionally, did the authors determine whether these aberrant mRNAs are stable? Could they be exported from the nucleus and possibly translated? A discussion regarding this aspect would be interesting since they mentioned “potential biological functions” of these transcripts in the Discussion.**

Thank you for this suggestion. We agree that this is a question of interest. However, since the aberrant mRNAs are unique to each clone, it will be necessary to study a large number of clones in detail to reach a clear conclusion on this point. We have added the following sentence to the Discussion section:
“To explore this possibility, further research is needed to test the stability and activity of these aberrant mRNAs.”


**3) Table 1: R and F legends are missing**

These have now been added to the Table 1 legend.

**4) Figure 2: In figure 2A, clone HA1 is also labelled clone 3.60. Figure 2B, C, D: 11.63 and 3.60 appear to have the same khaki color and cannot be discriminated; d2EGFP-11.50 is not visible.**

Thank you. The Fig 2A Labels are now corrected. **Figure 2B, C, D are updated with different colour code.**

**5) In S6 Fig, clone 11.60 is mentioned. Should it be clone 11.63?**

Thank you for spotting this. The label has been corrected to 11.63.

**References**

Davidson, I. F. and J. M. Peters (2021). "Genome folding through loop extrusion by SMC complexes." Nat Rev Mol Cell Biol **22**(7): 445-464.

Heinz, S., L. Texari, M. G. B. Hayes, M. Urbanowski, M. W. Chang, N. Givarkes, A. Rialdi, K. M. White, R. A. Albrecht, L. Pache, I. Marazzi, A. García-Sastre, M. L. Shaw and C. Benner (2018). "Transcription Elongation Can Affect Genome 3D Structure." Cell **174**(6): 1522-1536.e1522.

Kempfer, R. and A. Pombo (2020). "Methods for mapping 3D chromosome architecture." Nat Rev Genet **21**(4): 207-226.

Kiik, H., S. Ramanayake, M. Miura, Y. Tanaka, A. Melamed and C. R. M. Bangham (2022). "Time-course of host cell transcription during the HTLV-1 transcriptional burst." PLoS Pathog **18**(5): e1010387.

Lohia, R., N. Fox and J. Gillis (2022). "A global high-density chromatin interaction network reveals functional long-range and trans-chromosomal relationships." Genome Biol **23**(1): 238.

Melamed, A., H. Yaguchi, M. Miura, A. Witkover, T. W. Fitzgerald, E. Birney and C. R. Bangham (2018). "The human leukemia virus HTLV-1 alters the structure and transcription of host chromatin in cis." Elife **7**.

Niederer, H. A., D. J. Laydon, A. Melamed, M. Elemans, B. Asquith, M. Matsuoka and C. R. Bangham (2014). "HTLV-1 proviral integration sites differ between asymptomatic carriers and patients with HAM/TSP." Virol J **11**: 172.

Ramanayake, S., D. A. Moulding, Y. Tanaka, A. Singh and C. R. M. Bangham (2022). "Dynamics and consequences of the HTLV-1 proviral plus-strand burst." PLoS Pathog **18**(11): e1010774.
